# Reduced Renal Colonization and Enhanced Protection by Leptospiral Factor H Binding Proteins as a Multisubunit Vaccine against Leptospirosis in Hamsters

**DOI:** 10.3390/vaccines7030095

**Published:** 2019-08-22

**Authors:** Teerasit Techawiwattanaboon, Christophe Barnier-Quer, Tanapat Palaga, Alain Jacquet, Nicolas Collin, Noppadon Sangjun, Pat Komanee, Surapon Piboonpocanun, Kanitha Patarakul

**Affiliations:** 1 Department of Microbiology, Faculty of Medicine, Chulalongkorn University, Pathumwan, Bangkok 10330, Thailand; 2 Chula Vaccine Research Center (Chula VRC), Center of Excellence in Vaccine Research and Development, Chulalongkorn University, Pathumwan, Bangkok 10330, Thailand; 3 Vaccine Formulation Laboratory (VFL), University of Lausanne, Epalinges 1066, Switzerland; 4 Department of Microbiology, Faculty of Science, Chulalongkorn University, Bangkok 10330, Thailand; 5 Armed Force Research Institute of Medical Sciences (AFRIMS), Ratchathewi, Bangkok 10400, Thailand; 6 Institute of Molecular Biosciences, Mahidol University, Nakhon Pathom 73170, Thailand

**Keywords:** leptospirosis, *Leptospira*, factor H binding protein, multisubunit vaccine, neutral liposome, MPL, QS21

## Abstract

Subunit vaccines conferring complete protection against leptospirosis are not currently available. The interactions of factor H binding proteins (FHBPs) on pathogenic leptospires and host factor H are crucial for immune evasion by inhibition of complement-mediated killing. The inhibition of these interactions may be a potential strategy to clear leptospires in the host. This study aimed to evaluate a multisubunit vaccine composed of four known leptospiral FHBPs: LigA domain 7–13 (LigAc), LenA, LcpA, and Lsa23, for its protective efficacy in hamsters. The mono and multisubunit vaccines formulated with LMQ adjuvant, a combination of neutral liposome, monophosphoryl lipid A, and *Quillaja saponaria* fraction 21, induced high and comparable specific antibody (IgG) production against individual antigens. Hamsters immunized with the multisubunit vaccine showed 60% survival following the challenge by 20× LD_50_ of *Leptospira interrogans* serovar Pomona. No significant difference in survival rate and pathological findings of target organs was observed after vaccinations with multisubunit or mono-LigAc vaccines. However, the multisubunit vaccine significantly reduced leptospiral burden in surviving hamsters in comparison with the monosubunit vaccines. Therefore, the multisubunit vaccine conferred partial protection and reduced renal colonization against virulence *Leptospira* infection in hamsters. Our multisubunit formulation could represent a promising vaccine against leptospirosis.

## 1. Introduction

Leptospirosis is a worldwide zoonosis caused by pathogenic *Leptospira* spp. [1]. Currently available killed whole-cell vaccines for leptospirosis are mostly used in animals, but their use in humans has been limited because of short-term immunity, failure to cross-protect against a broad range of pathogenic serovars, and several adverse effects [2]. To overcome these limitations, subunit vaccines containing various leptospiral outer membrane proteins (OMPs), such as OmpL1, LipL32, LipL41, LemA, OmpA, OmpL37, and Loa22, have been tested [3,4,5,6,7,8]. So far, the C-terminal Ig-like domain 7–13 of LigA (LigAc) is presently the most promising vaccine candidate [9,10,11]. However, none of subunit vaccines confers sterilizing immunity.

After host entry, leptospires disseminate through bloodstream to target organs. In contrast with saprophytic species, most pathogenic leptospires evade complement-mediated killing [12] by interacting with factor H (FH) and C4b-binding protein (C4BP), the host complement negative regulators of the alternative pathway, and the classical and lectin pathways, respectively [13,14,15]. Factor H binding proteins (FHBPs) have been used as vaccine antigens against serogroup B meningococcal disease [16,17] and anti-FHBP antibodies could prevent FH binding to the bacteria leading to complement-mediated bacterial killing [18,19,20]. LigAc, LenA, LcpA, and Lsa23 are known conserved leptospiral FHBPs in pathogenic species, especially *L. interrogans*, and expressed during infection [15,19,21,22,23,24,25]. Therefore, we proposed that the combination of these FHBPs may enhance protection against leptospiral infection.

The present study aimed to determine the immunogenicity and protective efficacy of the multisubunit vaccine comprising four known FHBPs; LigAc, LenA, LcpA, and Lsa23; in golden Syrian hamsters, a widely accepted model of acute lethal leptospirosis. We showed the ability of this multisubunit vaccine formulation to reduce renal colonization of *Leptospira* in comparison with the monosubunit LigAc vaccine. In addition, our results support the idea of using a multisubunit vaccine design as an effective strategy to induce sterilizing immunity against pathogenic *Leptospira*.

## 2. Materials and Methods

### 2.1. Ethics Statement

All procedures involving manipulations of BALB/c mice were approved by the Institutional Animal Care and Use Committee (IACUC) of the Faculty of Medicine, Chulalongkorn University, Thailand (approved No. 015/2560). Mice were purchased from the National Laboratory Animal Center, Mahidol University, Thailand. All procedures involving manipulations of golden Syrian hamsters were approved by the IACUC of the Armed Forces Research Institute of Medical Sciences, Thailand (approved No. ARAC 1/60). Outbred hamsters were purchased from the Northeast Laboratory Animal Center, Khon Kaen University, Thailand. All procedures involving animals followed Thai National Animals for Scientific Purposes Act, BE 2558 (AD 2015), and were conducted under licenses issued by the Institute for Animals for Scientific Purpose Development and National Research Council of Thailand.

### 2.2. Bacterial Strains and Culture Conditions

Low-passage virulent *Leptospira interrogans* serovar Pomona (directly isolated from hamsters followed by < 5 in vitro passages) were used in all experiments [26]. Leptospires were cultured at 30 °C in Ellinghausen–McCullough–Johnson–Harris (EMJH) medium (BD Difco™, MD, USA) supplemented with 10% bovine serum albumin (BSA) [27]. *Escherichia coli* strains DH5α and BL21 (DE3) pLysS (Novagen, Darmstadt, Germany) were grown at 37 °C in Luria–Bertani (LB) medium with the addition of 100 µg/mL ampicillin and 30 µg/mL chloramphenicol when required.

### 2.3. LMQ Adjuvant Preparation

LMQ adjuvant, a combination of neutral liposome, monophosphoryl lipid A (MPL) and saponin from *Quillaja saponaria* (QS21), was prepared by the Vaccine Formulation Laboratory [28]. Briefly, neutral liposomes (2.5 mg/mL cholesterol and 10 mg/mL 1, 2-dioleoyl-*sn*-glycero-3-phosphocholine) were prepared by lipid film-rehydration and downsized by extrusion. A solution of MPL from *Salmonella enterica* serotype Minnesota (Sigma-Aldrich, St. Louis, MO, USA) and QS21 saponin were mixed with the liposome suspension in a 1:3 (*v*/*v*) ratio. The final volume ratio of LMQ to immunogen was 6:4.

### 2.4. Production of Recombinant FHBPs

Polymerase chain reaction (PCR) products encoding each FHBP without predicted signal sequence were amplified using genomic DNA of *L. interrogans* serovar Pomona as a template and the primers listed in Supplementary Table S1. The DNA amplicons encoding for the C-terminal portion corresponding to nucleotides 1892–3675 of LigA (NCBI reference sequence AE016823.1; locus_tag = LIC_10465), LcpA (LIC_11947), and Lsa23 (LIC_11360) were cloned into pRSET C (Invitrogen™, Carlsbad, CA, USA) at *Bam*HI and *Hind*III sites, while LenA (LIC_12906) amplicon was cloned into pET-15b (Novagen) at *Nde*I and *Xho*I sites. The recombinant plasmids were transformed into *E. coli* DH5α and verified by DNA sequencing (Macrogen Inc., Seoul, South Korea). The expression of recombinant proteins carrying the N-terminal 6× His tag was induced in *E. coli* BL21 (DE3) pLysS by addition of 1 mM isopropyl β-d-1-thiogalactopyranoside (IPTG) in the bacterial culture for 4 h at 37 °C. The cell pellets, subsequently resuspended in phosphate buffered saline (PBS) pH 7.4, were disrupted using a high-pressure homogenizer (Constant System Ltd., Northants, UK). Inclusion bodies, isolated by low speed centrifugation (3000× *g* for 30 min), were washed with washing buffer (0.5% Triton X-100, 1 M urea in PBS) and subsequently solubilized with denaturing buffer (6 M urea, 5 mM dithiothreitol (DTT) in PBS). The protein samples were purified by immobilized metal ion affinity chromatography using Ni Sepharose columns (GE Healthcare, Buckinghamshire, UK) under denaturing conditions and refolded by multistep dialysis using PBS or Tris buffers with the final buffers: LigAc in Tris buffer pH 8.0, LenA and LcpA in PBS pH 7.4, and Lsa23 in Tris buffer pH 12.0; as previously described [10,21,22,29]. The secondary structures of each refolded antigen were determined from their circular dichroism (CD) spectra [30].

### 2.5. Western Blotting

The purified recombinant proteins were characterized by SDS-PAGE and transferred to nitrocellulose membranes. Nonspecific binding sites on the membranes were blocked with 0.5% (*w*/*v*) BSA in PBS plus 0.05% Tween 20 (blocking buffer) before incubating the membranes with primary mouse anti-His tag monoclonal antibody (1:5000; KPL, MD, USA). Then, the membranes were incubated with goat alkaline phosphatase (AP)-conjugated anti-mouse IgG secondary antibody (1:5000; KPL). All incubations were performed for 1 h at room temperature. Anti-His tag immunoreactivity was detected using a 5-bromo-4-chloro-3-indolyl-phosphate/nitro blue tetrazolium (BCIP/NBT) Phosphatase Substrate System (KPL). The protein band intensity was measured using ImageJ software [31].

### 2.6. Mouse Immunization

Female BALB/c mice at 4–6 weeks old (*n* = 3 per group) were immunized subcutaneously three times at two-week intervals with 100 µL total volume of various vaccine formulations listed in Supplementary Table S2. The final volume ratios of LMQ and Freund’s (Sigma-Aldrich) adjuvants to immunogen (5 µg or 20 µg of each protein) were 6:4 and 1:1, respectively. Complete Freund’s adjuvant was used for the first immunization followed by incomplete adjuvant for the second and third immunizations. One week after each immunization, blood samples were collected at the submandibular venous plexus.

### 2.7. Hamster Immunization and Challenge with Leptospira

Female golden Syrian hamsters at 4–6 weeks old (*n* = 5 per group) were vaccinated subcutaneously three times at two-week intervals with 250 µL total volume of various vaccine formulations including the adjuvant control listed in Supplementary Table S3. The final volume ratio of LMQ adjuvant to immunogen (20 µg of each protein) was 6:4. One week before challenge, approximately 100 µL of blood samples were aseptically and carefully collected by cardiac puncture from hamsters under deep anesthesia with isoflurane [32,33].

The hamsters were challenged intraperitoneally with 20× LD_50_ (approximately 200 cells) [9,10] of low-passage *L. interrogans* serovar Pomona, homologous to the heat-killed vaccine, two weeks after the third immunization. The hamsters were weighed and monitored daily for end-point criteria, including loss of appetite, gait or breathing difficulty, prostration, ruffled fur, convulsion, failure to respond to stimuli or other moribund symptoms [9,34], and ≥20% weight loss. The hamsters that presented any of the end-point criteria or survived up to four weeks after challenge were humanely killed with an overdose of isoflurane and exsanguination. Then, blood and tissue samples were aseptically collected for histopathology and bacterial burden analysis.

### 2.8. FHBP-Specific Antibodies Detection

Antibody titers were measured by enzyme-linked immunosorbent assay (ELISA) as previously described [35] with some modifications. Each well of 96-well microtiter plates was coated with 100 µL of each recombinant protein (5 µg/mL), lysate of heat-killed leptospires (1 × 10^8^ cells/mL) or BSA (5 µg/mL). Nonspecific binding sites in the wells were blocked with the same blocking buffer as used in Western blotting and serial dilutions of individual sera (1:100–1:312,500) were incubated in the wells. Then, the plates were incubated with goat anti-mouse or goat anti-hamster IgG conjugated with horseradish peroxidase (HRP) (1:5000; KPL). The antigen/antibody complexes were detected using a 3,3′, 5,5′ tetramethylbenzidine (TMB) Substrate Reagent Set (BD Biosciences, Franklin Lakes, NJ, USA) according to manufacturer’s instructions. Absorbance by the chromophore was measured at 450 nm using Varioskan Flash Multimode Reader (Thermo Fisher Scientific, Vantaa, Finland) to calculate the antibody titers.

The levels of IgG subclasses in hamsters were also determined. The wells were coated with each recombinant protein, incubated, and resultant chromophore was detected as done for total IgG described above. The coated plates were primarily incubated with hamster sera (1:100–1:312,500), followed by mouse anti-hamster IgG1 or IgG2 conjugated with biotin (1:5000; BD Pharmingen, Franklin Lakes, NJ, USA) before finally incubated with Streptavidin HRP (1:5000; BD Pharmingen). 

### 2.9. Human FH Binding Assay

The wells of 96-well microtiter plates were coated with each recombinant protein, blocked, incubated, and resultant chromophore was detected as done for the ELISA except that the coated plates were incubated with 100 µL of purified human complement FH (10 µg/mL; Sigma-Aldrich). The bound FH was incubated with mouse anti-human complement FH (1:5000; Thermo Scientific), followed by goat anti-mouse IgG conjugated with HRP (1:5000; KPL) before detection of the chromophore.

### 2.10. Histopathology

Hamster tissue samples (lung, liver and kidney) were fixed in 10% formalin buffer and embedded in paraffin. Subsequently, the samples were sectioned at 5 µm thickness and stained with hematoxylin and eosin. Histopathology was conducted by a board-certified veterinary pathologist who was blinded to experimental groups as described previously [36]. Pulmonary hemorrhage was graded as 0 (none), 1 (single focus), 2 (multivalent foci), or 3 (severe). Tubulointerstitial nephritis was assessed as 0 (normal), 1 (mild), 2 (moderate), or 3 (severe). Liver pathology was graded based on the average number of inflammatory foci in 10 fields at 10× magnification as 0 (none), 1 (1–3), 2 (4–7), or 3 (>7).

### 2.11. Leptospira Detection in Hamsters

About half of kidney volume was sliced into small pieces and then pulverized by passing them through 5 mL syringe and then inoculated into semisolid EMJH medium. Total DNA was extracted from kidneys using High Pure PCR Template Preparation Kit (Roche, Mannheim, Germany) according to manufacturer’s instructions for quantitative real-time PCR (qPCR) detection. The PCR was performed using Power SYBR Green PCR Master Mix (Applied Biosystem, Foster, CA, USA) with specific primers for leptospiral *lipL32* [37] and QuantStudio 5 Real-Time PCR System (Applied Biosystem) according to manufacturer’s instructions. Leptospiral DNA standard curve was constructed from ten-fold serially diluted DNA of *L. interrogans* serovar Pomona equivalent to 2 × 10^1^ to 2 × 10^9^ cells/mL.

### 2.12. Statistical Analysis

All statistical analyses were performed using IBM SPSS Statistics for Windows (version 22, Chulalongkorn University license). Survival and mortality for each group was examined using Kaplan–Meier curves and their differences were tested using a log-rank test. The significance of differences between antibody titers, histopathology score, and bacterial burdens were determined using a Mann–Whitney *U* test.

## 3. Results

### 3.1. Preparation and Characterization of Recombinant FHBPs

**Figure 1:**
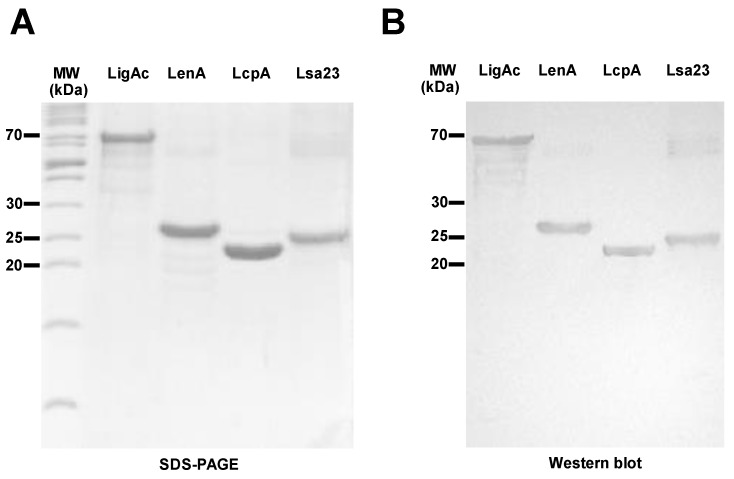

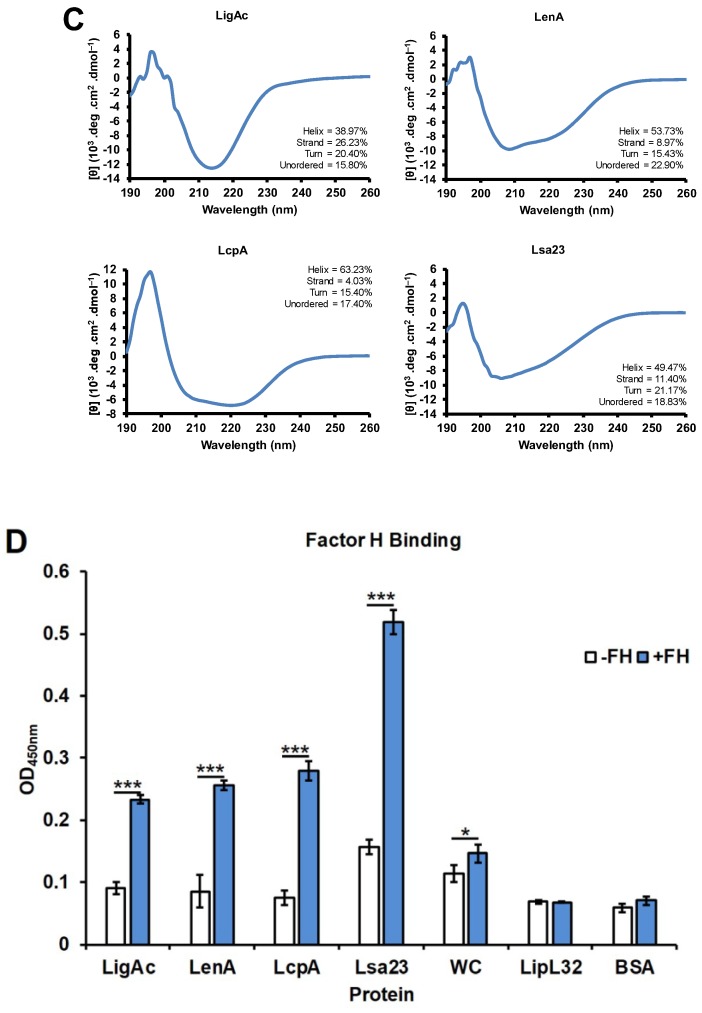
The recombinant factor H binding proteins (FHBPs: LigAc, LenA, LcpA, and Lsa23). (**A**) Purified recombinant FHBPs were subjected to 12% SDS-PAGE under reducing conditions and stained with Coomassie Brilliant Blue R-250; (**B**) The separated recombinant proteins were blotted onto nitrocellulose membranes and detected with mouse anti-His tag monoclonal antibody (primary antibody) and goat alkaline phosphatase-conjugated anti-mouse (secondary antibody) and its reaction with the BCIP/NBT Phosphatase Substrate System. The positions of PageRuler Unstained Protein Ladder (Thermo Fisher Scientific) are indicated to the left; (**C**) CD spectra of recombinant FHBPs measured using a JASCO J-815-150S spectropolarimeter and analyzed with CDPro program. CD spectra are represented as an average of more than five spectra from 190 to 260 nm; (**D**) Binding of recombinant FHBPs to purified human FH. The results are shown as mean ± SD absorbance at 450 nm from three independent human FH binding assays. * represents *p* < 0.05 and *** represents *p* < 0.001 (independent two-sided Student *t*-tests). A whole cell lysate (WC) of leptospires was used as a positive control and recombinant LipL32 and bovine serum albumin (BSA) were used as negative controls.

Purified recombinant LigAc, LenA, LcpA, and Lsa23 proteins were detected at their expected molecular masses of 66.3, 25.4, 23.6, and 25.3 kDa, respectively, by SDS-PAGE and immunoblotting (Figure 1A,B). Their purities were more than 95% as determined by band intensities on SDS-PAGE using ImageJ software. Although all four recombinant FHBPs were initially expressed as inclusion bodies and needed to be purified under strong denaturing conditions, they were soluble after refolding by stepwise dialysis. Their secondary structures demonstrated by CD spectra (Figure 1C) and the ability to bind to human FH (Figure 1D) suggested that the purified recombinant FHBPs were properly refolded and retained the FH binding activity before using as vaccine antigens.

### 3.2. Immunogenicity of Mono or Multisubunit FHBP Vaccines in Mice

**Figure 2:**
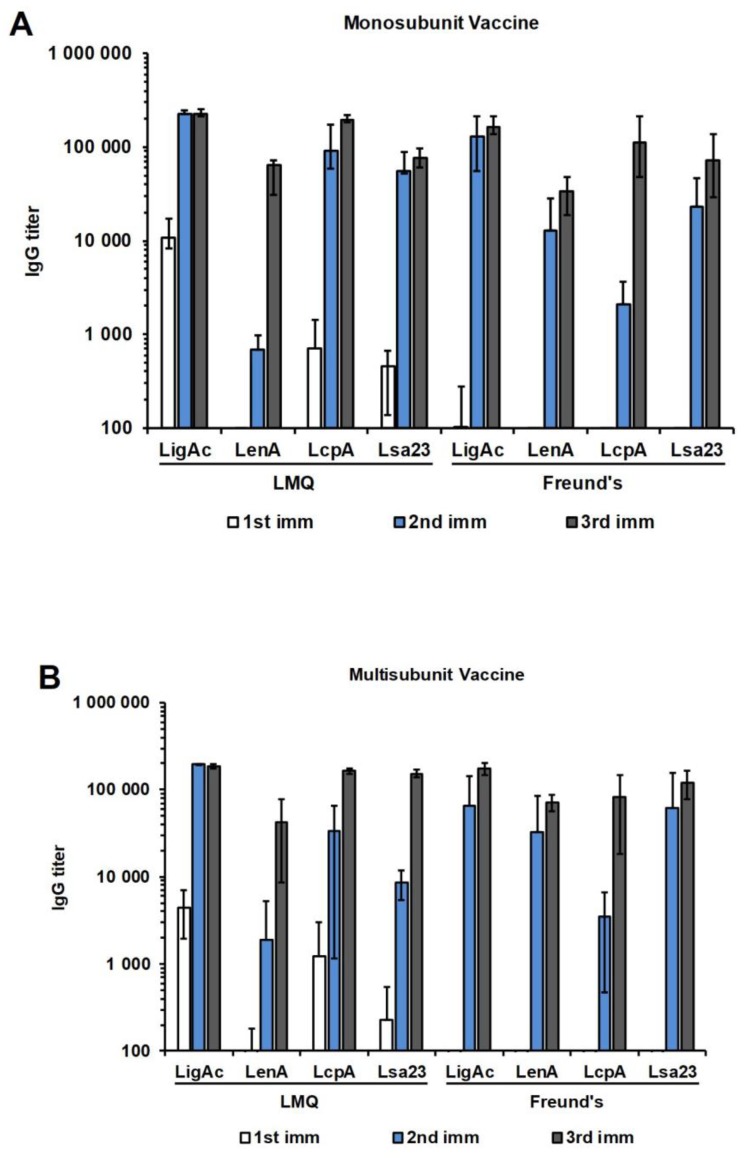
Antibody levels in mice immunized with various recombinant factor H binding protein (FHBP) subunit vaccine formulations. The vaccines were formulated with either LMQ (a combination of neutral liposome, monophosphoryl lipid A, and *Quillaja saponaria* fraction 21) or Freund’s adjuvants. The antibody titers at one week after each immunization (imm) were measured by enzyme-linked immunosorbent assay (ELISA). (**A**) Antibody titers after immunization with individual recombinant proteins as mono-FHBP (LigAc, LenA, LcpA, and Lsa23) subunit vaccines. (**B**) Antibody titers after immunization with pooled recombinant FHBPs as a multisubunit vaccine. The specific antibody titers to tested antigens shown (as bars) were determined by subtracting the titers for nonspecific reactivity to recombinant 6× His tag non-FHBP (recombinant LipL32) and BSA from the total titers. The results are shown as mean ± SD.

All formulations induced significantly higher specific antibody levels than the adjuvant control containing the LMQ adjuvant mixed with PBS without vaccine antigens (of which the antibody level was comparable to that of background in mice). The antibody titers against each FHBP reached a maximum level after three immunizations and were comparable between the groups using LMQ and Freund’s adjuvants (Figure 2A). The combination of four FHBPs as the multisubunit vaccine did not antagonize or enhance the immunogenicity of any individual antigen (Figure 2B).

In addition, we used LcpA as a representative antigen to test two different doses (5 and 20 μg per mouse) of antigen in combination with either of two different adjuvants (LMQ and Freund’s) to compare the immunogenicity of different vaccine formulations. We found no significant difference of antibody titers between the two doses after three immunizations (Supplementary Figure S1). Similarly, LMQ adjuvant was as effective as Freund’s adjuvant in inducing antibody production.

### 3.3. Immunogenicity of Mono or Multisubunit FHBPs in Hamsters

**Figure 3:**
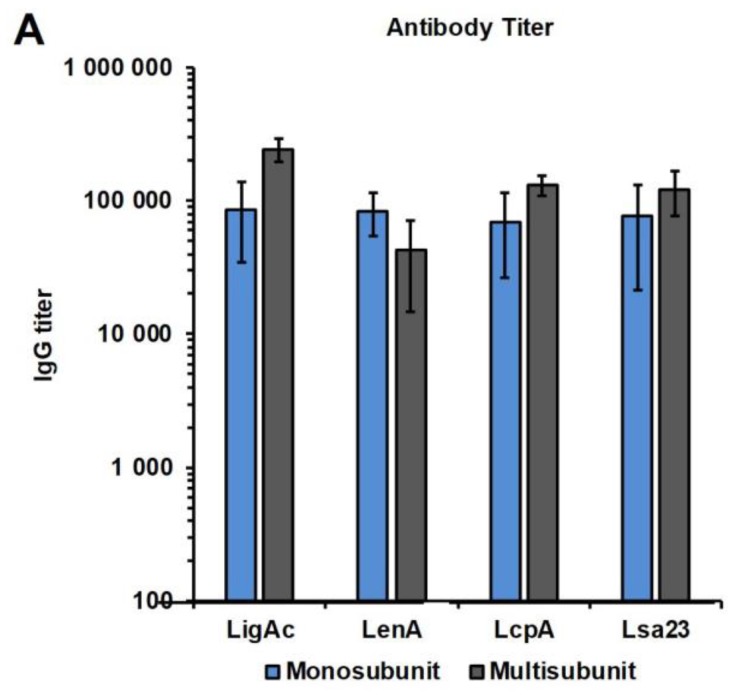

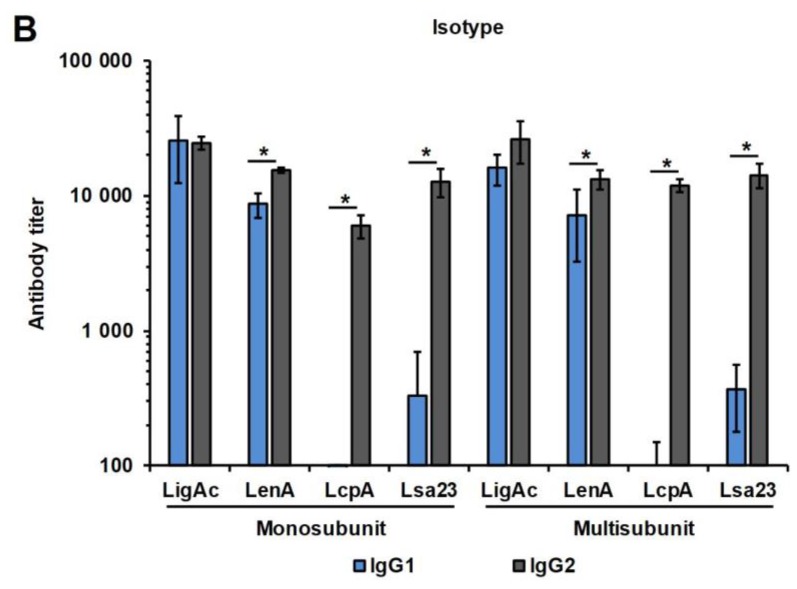
Antibody levels in hamsters immunized with monosubunit vaccines or vaccines containing multiple subunits of recombinant factor H binding proteins (FHBPs). The vaccines were formulated with LMQ (a combination of neutral liposome, monophosphoryl lipid A, and *Quillaja saponaria* fraction 21). The antibody titers at one week after the third immunization were measured by enzyme-linked immunosorbent assay (ELISA). (**A**) Antibody titers after immunizations with individual recombinant proteins as mono-FHBP vaccine or pooled recombinant FHBPs as a multisubunit vaccine; (**B**) Isotyping of anti-LigAc, LenA, LcpA, and Lsa23 IgG subclasses. The specific antibody titers to tested antigens shown (as bars) were determined by subtracting the titers for nonspecific reactivity to recombinant 6× His tag non-FHBP (recombinant LipL32) and BSA from the total titers. The results are shown as mean ± SD. Mann–Whitney *U* test was used to compare antibody titer among vaccination groups; * represents *p* < 0.05.

After immunizing the hamsters three times, the antibody levels against each recombinant FHBP were determined one week after the third immunization, which was one week before the challenge (Figure 3). The high specific antibody titers to all immunogens were found in hamsters vaccinated with both mono and multisubunit vaccines combined with LMQ (Figure 3A). These results demonstrated that individual recombinant FHBP and the combination of four FHBPs at 20 µg of each antigen were immunogenic in hamsters, consistent with the results obtained in mice. In addition, the IgG subclasses in hamsters induced by each FHBP vaccine formulation were evaluated. LigAc formulation induced comparable levels of IgG1 and IgG2, whereas LenA, LcpA, and Lsa23 formulations induced significantly higher IgG2 than IgG1 (*p* < 0.05) (Figure 3B). The IgG subclass profiles induced by each vaccine antigen were not significantly different between the mono and multisubunit vaccines. In addition, there was no significant difference in the isotype levels between survivors and non-survivors of each vaccine antigen (data not shown).

It should be noted that all hamsters were closely monitored after blood collection and showed full recovery with no hamster death before challenge. No gross cardiac or mediastinal hematoma and hemorrhage was observed in all hamsters when they were sacrificed.

### 3.4. Protective Efficacy of Multisubunit FHBP Vaccine in Hamsters

**Figure 4:**
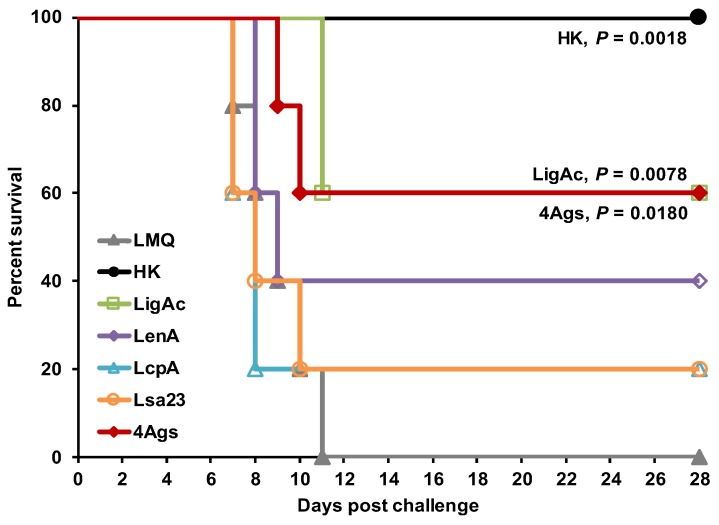
Kaplan–Meier plot showing protection of vaccinated hamsters against challenge by live *Leptospira*. The hamsters were immunized with the various vaccine formulations shown in Table S3. Each vaccinated hamster was challenged intraperitoneally by 20× LD_50_ of low passage leptospires. Their survival was monitored daily until the end point of 28 days. The percentage of hamster survival was calculated as the number of survivors divided by the total number of animals challenged. Statistical values of survival rate between adjuvant control group and other vaccination groups were analyzed by log-rank test. Vaccination was with LMQ = phosphate buffered saline and LMQ (a combination of neutral liposome, monophosphoryl lipid A, and *Quillaja saponaria* fraction 21); HK = Heat-killed whole cell vaccine; LigAc, LenA, LcpA or Lsa23 = LigAc, LenA, LcpA, or Lsa23 mono recombinant factor H binding protein (FHBP) and LMQ; 4Ags = multiple recombinant FHBPs (LigAc + LenA + LcpA + Lsa23) and LMQ.

Vaccinated hamsters were challenged with virulent leptospires to evaluate the protective efficacy of the FHBP subunit vaccines (Figure 4 and Table 1). The immunization with monosubunit vaccine containing LigAc, LenA, LcpA, and Lsa23 individually conferred 60%, 40%, 20%, and 20% survival, respectively. The survival rate of hamsters immunized with the multisubunit vaccine (60%, *p* = 0.018) was significantly higher than that of hamsters in the adjuvant control group (0%) but was not significantly different from the group vaccinated with LigAc alone (60%, *p* = 0.850). As expected, control hamsters vaccinated with killed whole cell vaccines showed 100% survival after challenge with the homologous serovar, while all hamsters in the adjuvant control group died within two weeks.

### 3.5. Effect of Multisubunit FHBP Vaccine on Target Organ Invasion by Leptospira

Histopathological changes compatible with clinical features of leptospirosis indicated target organ involvement in challenged hamsters. Pulmonary hemorrhages with small foci and mild liver inflammation were observed in all groups but a high severity was found in non-survivors, especially in the lungs of hamsters vaccinated with LenA and LcpA (Table 2 and Supplementary Figures S2 and S3). Animals vaccinated with the killed whole cell vaccines exhibited no tubulointerstitial nephritis contrasting with that of FHBP subunit vaccines which presented mild kidney injury (Supplementary Figure S4). Although hamsters vaccinated with the LigAc monosubunit vaccine seemed to have lower pathological scores than those received the multisubunit vaccine, there was no significant difference between these two groups.

**Figure 5:**
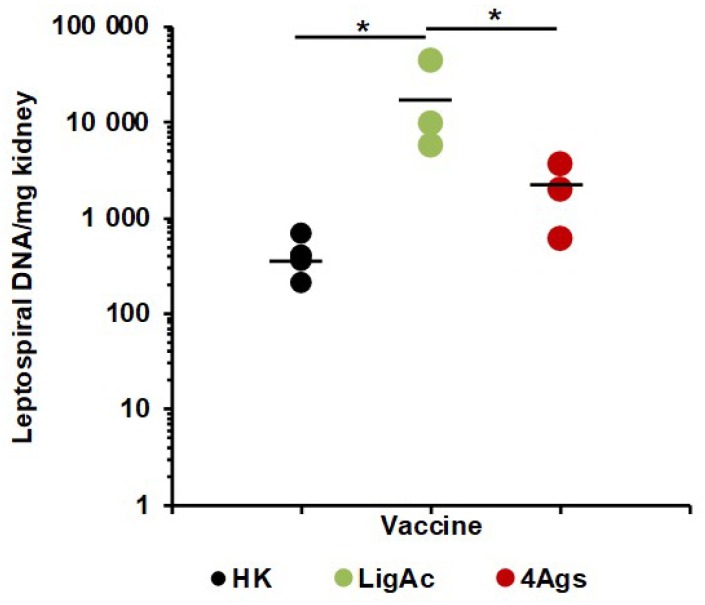
Leptospiral load in kidneys of surviving hamsters immunized with mono-LigAc subunit vaccine or vaccines containing multiple subunits of recombinant factor H binding proteins (FHBPs). Total genomic DNA was extracted from kidneys and detected by qPCR. The cycle threshold of each sample was compared with leptospiral DNA standard curve to calculate bacterial burden which is expressed as bacterial DNA per milligram of tissue. The results are shown as mean of each vaccination group; HK = Heat-killed whole cell vaccine; LigAc = LigAc mono recombinant factor H binding protein (FHBP) and LMQ; 4Ags = multiple recombinant FHBPs (LigAc + LenA + LcpA + Lsa23) and LMQ. Only the HK, LigAc, 4Ags groups with at least 3 survivors were used for statistical analysis. Mann–Whitney *U* test was used to compare bacterial number among vaccination groups; * represents *p* < 0.05.

We detected *Leptospira* in the kidneys of surviving hamsters to provide evidence for sterilizing immunity (Figure 5 and Table 1). In all hamsters that viable leptospires were not found in the kidneys by culture, leptospiral DNA were detected by qPCR. Kidneys from hamsters immunized with the LigAc vaccine had a significant higher leptospiral burden than those immunized with the heat-killed vaccines (*p* < 0.05) (Figure 5). More importantly, the multisubunit vaccine significantly reduced leptospiral load more than the LigAc vaccine (*p* < 0.05). There was no significant difference in leptospiral burden in the kidneys of the multisubunit vaccinated and the heat-killed groups. Additionally, the analysis using either only survivors or all hamsters came to a similar conclusion. These results indicate that the combination of four FHBPs reduced renal colonization against leptospiral infection.

Pulmonary hemorrhage was graded as 0 (none), 1 (single focus), 2 (multivalent foci), or 3 (severe). Tubulointerstitial nephritis was assessed as 0 (normal), 1 (mild), 2 (moderate), or 3 (severe). Liver pathology was graded based on the average number of inflammatory foci in 10 fields at 10× magnification as 0 (none), 1 (1–3), 2 (4–7), or 3 (>7).

## 4. Discussion

Binding of FHBPs on pathogenic *Leptospira* to host FH is a crucial strategy for evasion of complement-mediated killing to survive and establish the infection [12,15,19]. Antibodies against FHBPs may inhibit FH binding to pathogenic leptospires and enhance the susceptibility to complement lysis. Several leptospiral OMPs, such as LenA (or LfhA) [24], LcpA [15], and Lsa23 [19], have been identified as FHBPs. A previous study showed that pathogenic *L. interrogans* became more susceptible to complement killing after blocking with anti-Lsa23 inactivated serum [19]. We hypothesized that multiple FHBPs including LigAc, LenA, LcpA, and Lsa23, in the multisubunit vaccine could synergistically enhance the protective immune response against leptospirosis. 

Any protein-based subunit vaccine needs to be formulated with adjuvants to drastically increase the immunogenicity. In previous studies, Freund’s adjuvant was used with LigA and it was reported to provide high survival of hamsters [9,38]. However, this adjuvant can induce adverse side effects and currently poses ethical issues for both human and animal use. The widely used alum adjuvant has been tested for leptospirosis vaccines with equivocal results [4,11,36,39]. *Salmonella* flagellin was previously shown as the only adjuvant in the LigAc-based vaccine formulations that were able to reduce leptospiral renal colonization in hamsters [11]. Several studies demonstrated the advantage of liposome-based adjuvants in promoting an immune response to various pathogens [40,41,42] and stimulating immune responses in populations that responded insufficiently to alum [43,44,45]. Therefore, the present study used an adjuvant called LMQ [28], a combination of neutral liposomes, the toll-like receptor 4 agonist MPL [46], and the Saponin QS-21 [47], to induce high antibody levels against each antigen in the multisubunit vaccine.

Initially, we evaluated the immunogenicity of single and multiple antigens administered with LMQ or Freund’s adjuvant in BALB/c mice. These adjuvants were able to induce comparable levels of specific antibodies against each FHBP in mice immunized with either the mono or multisubunit vaccine (Figure 2). After three immunizations, there was no difference in antibody titers between two tested doses (5 and 20 μg) (Supplementary Figure S1). Therefore, hamsters (weighing four–five times more than BALB/c mice) were immunized with 20 μg of each vaccine antigen with LMQ. As expected, all recombinant FHBPs induced high antibody titers in hamsters (Figure 3).

Unlike previous reports [9,48,49], our separate studies in hamsters showed that 20%, but not 10%, weight loss was correlated with dead outcome (Supplementary Figure S5). The hamsters with 10% weight loss could later gain their weight and finally survived up to the end of the experiment. It is possible that different *Leptospira* strains and the source of hamsters may affect the challenge outcome. Therefore, we set 20% weight loss as one of the end point criteria before the challenge. However, in this study, no hamsters met this endpoint criterion because all non-surviving hamsters died with moribund symptoms before reaching 20% weight loss.

The vaccine formulations have a critical impact on their protective efficacy as shown in hamsters immunized with various LigAc vaccine formulations using different doses and adjuvants [11,36,39,50]. In previous studies of the LigAc subunit vaccine against lethal leptospirosis in hamsters, the highest protection (100% survival) was conferred by three immunizations with 100 µg of LigAc formulated with Freund’s adjuvant [9,38] in comparison to the controls receiving PBS mixed with the same adjuvant (0% survival). The survival of hamsters immunized with two doses of LigAc and Freund’s adjuvant was 63% (20/10 µg) to 80–100% (80/40 µg) [10]. In the present study, we immunized the hamsters with 20 µg of each antigen because of the maximal concentration of antigens (1 mg/mL) and the limits to the total volume that could be delivered subcutaneously. Moreover, as we aimed to observe any potential synergistic effect of three FHBPs on LigAc vaccination, LigAc overdose up to 100 µg could mask these effects. Our vaccine formulation with 20 µg of LigAc administrated with LMQ conferred the partial protective efficacy (60% survival) as comparable as reported by other groups [10,36]. The results highlighted that LMQ could be a promising adjuvant for future development of effective leptospirosis subunit vaccines.

The IgG subclass profiles in hamsters were determined to identify their correlation with protection (Figure 3B). IgG3 was not included for detection because it is not expressed in all hamster strains and has not yet been shown to correlate with Th1/Th2 profile [51]. Because the same LMQ adjuvant and administration strategy were applied for all vaccine formulations, the IgG subclass profiles most likely depends on the vaccine antigen preparations. Only LigAc formulation induced equal levels of IgG1 and IgG2 suggesting a balanced Th1/Th2 immune response. Significantly higher levels of IgG2 than IgG1 observed in LenA and predominant IgG2 levels induced by LcpA and Lsa23 formulations indicate mainly a Th1 response. However, survivors and non-survivors showed no significant difference of IgG isotype levels for all vaccine formulations. Therefore, no predominant IgG isotype or Th immune response was correlated with protective efficacy of each FHBP formulation in either the mono or multisubunit vaccines. In some studies, IgG2 isotype or Th1 response has been observed to confer protection against leptospirosis [34,52,53]. However, Th2 is conventionally believed to be responsible for protection against extracellular pathogens including leptospires in spite of their transient tissue invasion [54]. Further investigations are required to elucidate the role of isotype or Th response in protection and the vaccine efficacy against pathogenic *Leptospira* spp.

The protective efficacy of the multisubunit FHBP vaccine adjuvanted with LMQ for survival was not different from the partial rate that obtained from the mono-LigAc subunit vaccine (Figure 4), therefore LigAc is most likely the key vaccine antigen of the multisubunit vaccine. However, renal colonization of leptospires was significantly reduced in the surviving hamsters (Figure 5). Similar to the previous study [9], the mono-LigAc subunit vaccine did not reduce renal colonization compared to the adjuvant control (Figure 5). According to a good quality of all vaccine antigens as shown by their integrity on SDS-PAGE (Figure 1A) and immunoblotting (Figure 1B), secondary structures by CD spectrum analysis (Figure 1C), ability to bind FH (Figure 1D), and immunogenicity (Figure 3A), they could involve in these positive effects together, but a prominent antigen is still unclear. 

Although the multisubunit FHBP vaccine was unable to induce sterilizing immunity, it significantly reduced renal colonization in survivors after challenge compared with the LigAc monosubunit vaccines but was not different from the heat-killed group. It is possible that addition of three more FHBPs to LigAc in the multisubunit vaccine might induce protective antibodies that conferred more complement killing of leptospires resulting in reduced renal colonization. Moreover, these vaccine antigens are actually multifunctional proteins, interacting with not only FH but also C4BP [15,19,22,55], host extracellular matrix (ECM) proteins such as vitronectin [15], fibronectin and laminin [29,56], and plasminogen [19,57,58]. Hence, the antibody production triggered by this multisubunit vaccine may induce synergistic effect on protection. The synergistic effects against leptospirosis have previously been reported for other antigens, such as the combination of OmpL1 and LipL41 [3], and three probable OMPs of Lp1454, Lp1118, and MceII [59]. 

Previously only flagellin-based LigAc vaccines showed reduction of renal colonization which was evaluated by culture and microscopic examination of leptospires in silver-stained tissue sections [11]. These methods might not be able to detect very low number of leptospires in the renal tissues resulting in false negative results. In our study, we could not detect leptospires in the kidneys of survivors by culture (Table 1). Therefore, more sensitive qPCR was used to determine leptospiral burden in the kidneys in this study. Leptospiral DNA was detected by qPCR indicating leptospiral invasion in the kidneys of all survivors (Table 1). Surprisingly, no cultures were grown from the kidneys of the adjuvant controls. All hamsters in this group reached the endpoint criteria within 11 days (7–11 days) after challenge (Table 1). It is possible that they were in the late leptospiremic phase, therefore only a small number of leptospires had colonized the kidneys, which was too low to be detected by culture but was able to detect by more sensitive qPCR. Alternatively, a previous study on LemA and Erp Y-like subunit vaccines also showed negative kidney cultures in all hamsters survived up to 30 days post-challenge in spite of histopathological change [60]. The authors suggested that strong activation of the immune system not only resulted in renal damage but also cleared viable *Leptospira* in the kidney. Because qPCR could not differentiate live or dead bacteria, positive qPCR of the culture-negative kidney tissue from the controls might be a result of dead leptospires. However, the degree of pathological lesions in that study was more severe than that in our study. The discrepancy between pathological scores of liver and kidney injuries was observed between the adjuvant control and the mono-LigAc vaccine groups (Table 2). Previous studies showed distinct pathological changes of target organs in acute and chronic leptospirosis in animal models [61]. The controls reached the endpoint criteria within 11 days post-challenge, whereas hamsters immunized with the LigAc vaccine reached the endpoint criteria 11–28 days post-challenge. Therefore, the hamsters in each group were probably in different phases of disease resulting in their distinct histopathological changes.

In this study, we aimed to test the vaccine efficacy of four recombinant FHBPs in combination and individually, thus we do not know which one of three new FHBPs are responsible for enhanced reduction of renal colonization, which is the limitation of this study. Further investigations using different combinations of these subunit vaccines including all FHBPs, except LigAc, are required to determine the crucial components. Chimeric vaccines containing fusion of protective FHBPs will be a promising strategy to deliver the vaccine antigens as previously reported [49,52,53].

## 5. Conclusions

The multisubunit vaccine consisting of four leptospiral FHBPs; LigAc, LenA, LcpA, and Lsa23, with LMQ adjuvant conferred partial protection against the lethal challenge with *L. interrogans* serovar Pomona in hamsters. The survival protection was most likely elicited by the effect of LigAc. The addition of three FHBPs to LigAc vaccine significantly reduced the leptospiral invasion in the kidneys. This study may lead to the development of improved vaccines for leptospirosis in humans who cannot be effectively immunized using available killed whole-cell vaccines because of their limitations.

## Figures and Tables

**Table 1:** Protection conferred by immunization with vaccines in hamsters.

Vaccine Formulation ^a^		% Protection ^b^	Endpoint Day	Kidney Positive Detection ^c^	
Antigen	Adjuvant			Culture	qPCR
PBS	LMQ	0	7, 8, 9, 10, 11	0/5	5/5
Heat-killed *Leptospira*	Freund’s	100 **	28, 28, 28, 28, 28	0/5	5/5
LigAc	LMQ	60 **	11, 11, 28, 28, 28	0/5	5/5
LenA	LMQ	40	8, 8, 9, 28, 28	0/5	5/5
LcpA	LMQ	20	7, 7, 8, 8, 28	0/5	5/5
Lsa23	LMQ	20	7, 7, 8, 10, 28	0/5	5/5
LigAc + LenA + LcpA + Lsa23	LMQ	60 *	9, 10, 28, 28, 28	0/5	5/5

^a^ PBS: phosphate buffered saline; LMQ: a combination of neutral liposome, monophosphoryl lipid A, and *Quillaja saponaria* fraction 21. LigAc, LenA, LcpA, Lsa23: recombinant factor H binding protein subunit vaccines. The final volume ratio of LMQ or Freund’s adjuvants to immunogen was 6:4 or 1:1, respectively. ^b^ % Protection was calculated by the number of survivors/total challenged hamsters ×100 (*n* = 5/group). Statistical values of survival rate between adjuvant control group and other vaccination groups were analyzed by log-rank test; * represents *p* < 0.05 and ** represents *p* < 0.01. ^c^ Positive detection was evaluated in all hamsters by culture and/or real-time PCR techniques. The results showed the number of positive detection/total challenged hamsters. The PCR negative detection for leptospires was assigned in a sample whose threshold cycle value was greater than 40 cycles.

**Table 2:** Pathology score conferred by immunization with vaccines in hamsters.

Vaccine Formulation ^a^		Mean Pathological Score ^b^		
Antigen	Adjuvant	Lung	Liver	Kidney
PBS	LMQ	0.8 ± 1.1	1.0 ± 1.4	0.8 ± 0.5
Heat-killed *Leptospira*	Freund’s	0.6 ± 0.6	1.0 ± 0.0	0.0 ± 0.0 *
LigAc	LMQ	0.6 ± 0.9	0.4 ± 0.6	1.2 ± 0.8
LenA	LMQ	1.4 ± 1.3	1.0 ± 0.7	0.4 ± 0.9
LcpA	LMQ	2.0 ± 1.2	1.2 ± 0.4	0.4 ± 0.6
Lsa23	LMQ	1.2 ± 0.8	0.8 ± 0.5	1.0 ± 0.7
LigAc, LenA, LcpA, Lsa23	LMQ	1.2 ± 0.6	1.2 ± 0.6	1.0 ± 1.0

^a^ PBS: phosphate buffer saline; LMQ: a combination of neutral liposome, monophosphoryl lipid A, and *Quillaja saponaria* fraction 21. LigAc, LenA, LcpA, Lsa23: recombinant factor H binding protein subunit vaccines. The final volume ratio of LMQ or Freund’s adjuvants to immunogen was 6:4 or 1:1, respectively. ^b^ The histopathological scores were determined in all hamsters (*n* = 5/group) by a pathologist with blinding protocol. Statistical values of pathological score between adjuvant control group and other vaccination groups were analyzed by Mann–Whitney *U*-Test; * represents *p* < 0.05.

## Supplementary Materials

The following are available online at https://www.mdpi.com/2076-393X/7/3/95/s1. Figure S1: Antibody levels in mice immunized with mono-LcpA subunit vaccine formulated with different doses and adjuvants, Figure S2: Lung histopathology showing small foci from hamsters vaccinated with various vaccine formulations (Table S3), Figure S3: Liver histopathology showing mild inflammation in hamsters vaccinated with various vaccine formulations (Table S3), Figure S4: Kidney histopathology showing sparse tubulointerstitial nephritis from hamsters vaccinated with various vaccine formulations (Table S3), Figure S5: Weight of five surviving hamsters after challenge with virulence *L. interrogans* serovar Pomona, Table S1: Oligonucleotides used in this study, Table S2: Groups of mice immunized with various vaccine formulations containing recombinant factor H binding protein subunits or controls, Table S3: Groups of hamsters immunized with various vaccine formulations containing recombinant factor H binding protein subunits or controls.
