# Supplementary material for: Reduced Renal Colonization and Enhanced Protection by Leptospiral Factor H Binding Proteins as a Multisubunit Vaccine against Leptospirosis in Hamsters
Source: Vaccines (Basel). 2019 Aug 22;7(3):95. doi: 10.3390/vaccines7030095 (PMC6789851; doi:10.3390/vaccines7030095)
Supplement: Supplementary file 1 [file vaccines-07-00095-s001.pdf]

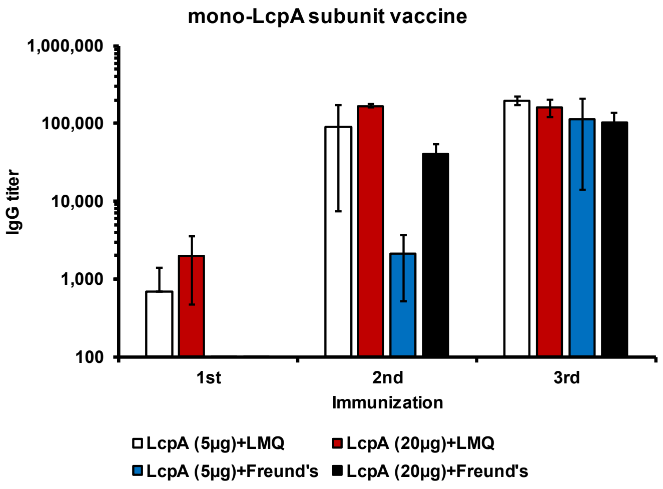

Figure S1. Antibody levels in mice immunized with mono-LcpA subunit vaccine formulated with different doses and adjuvants. Antibody levels in mice immunized with various doses (either 5µg or 20 µg per injection) of mono-LcpA subunit vaccine formulated with either LMQ (a combination of neutral liposome, monophosphoryl lipid A, and *Quillaja saponaria* fraction 21) or Freund's adjuvants. The antibody titers at one week after each immunization were measured by ELISA. The specific antibody titers to tested antigens shown (as bars) were determined by subtracting the titers for nonspecific reactivity to recombinant 6× His tag non-FHBP (recombinant LipL32) and BSA from the total titers. The results are shown as mean ± SD.#

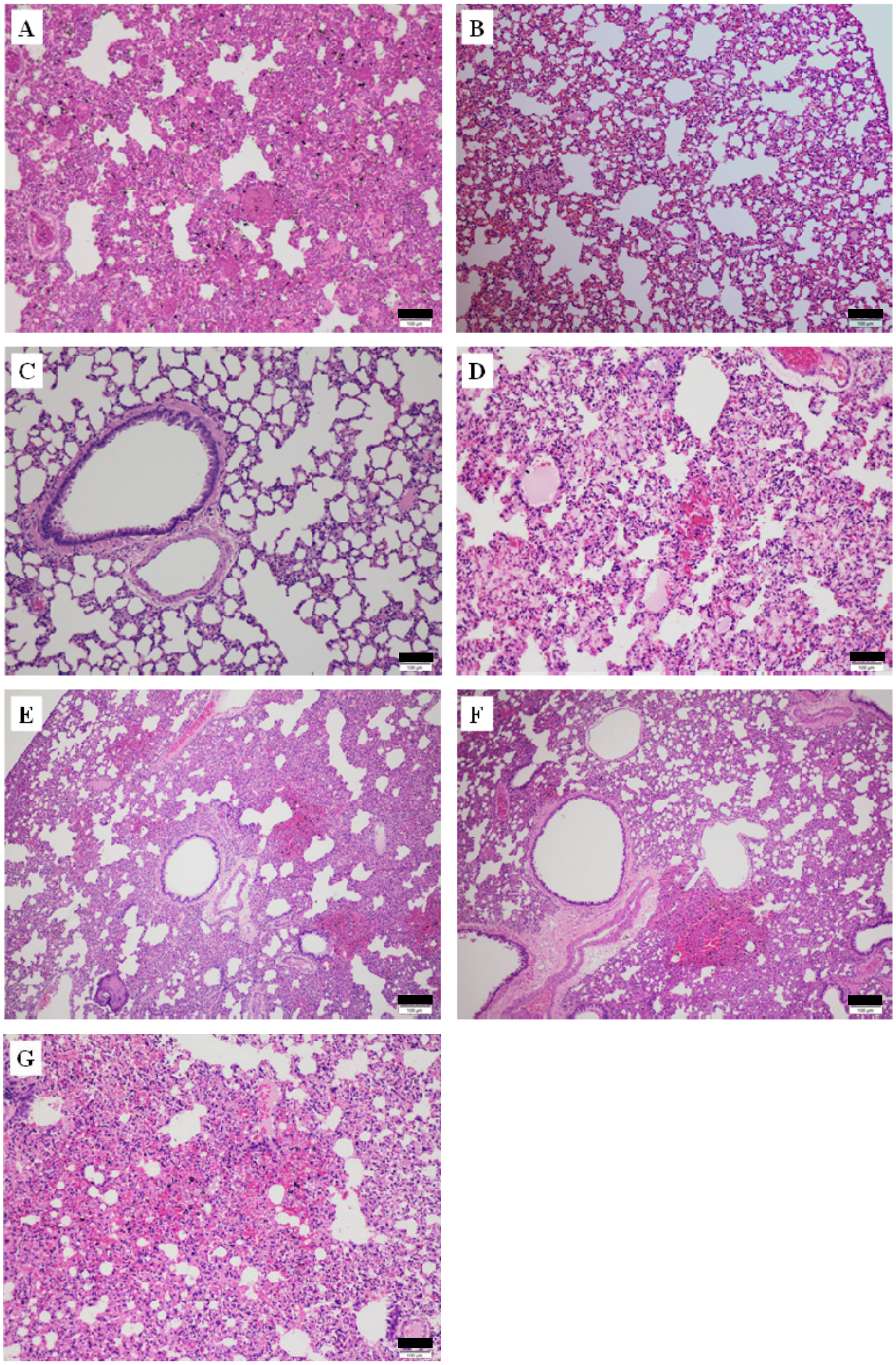

Figure S2. Lung histopathology showing small foci from hamsters vaccinated with various vaccine formulations (Table S3). The micrographs were recorded at 100× magnifications after hematoxylin and eosin staining. (**A**) LMQ (a combination of neutral liposome, monophosphoryl lipid A, and *Quillaja saponaria* fraction 21) adjuvant control; (**B**) Killed whole cell vaccine with Freund's adjuvant; (**C**) Mono-LigAc subunit vaccine with LMQ; (**D**) Mono-LenA subunit vaccine with LMQ; (**E**) Mono-LcpA subunit vaccine with LMQ; (**F**) Mono-Lsa23 subunit vaccine with LMQ; (**G**) Multisubunit vaccine (containing

LigAc, LenA, LcpA, and Lsa23) with LMQ. Black bar: 100 μm.

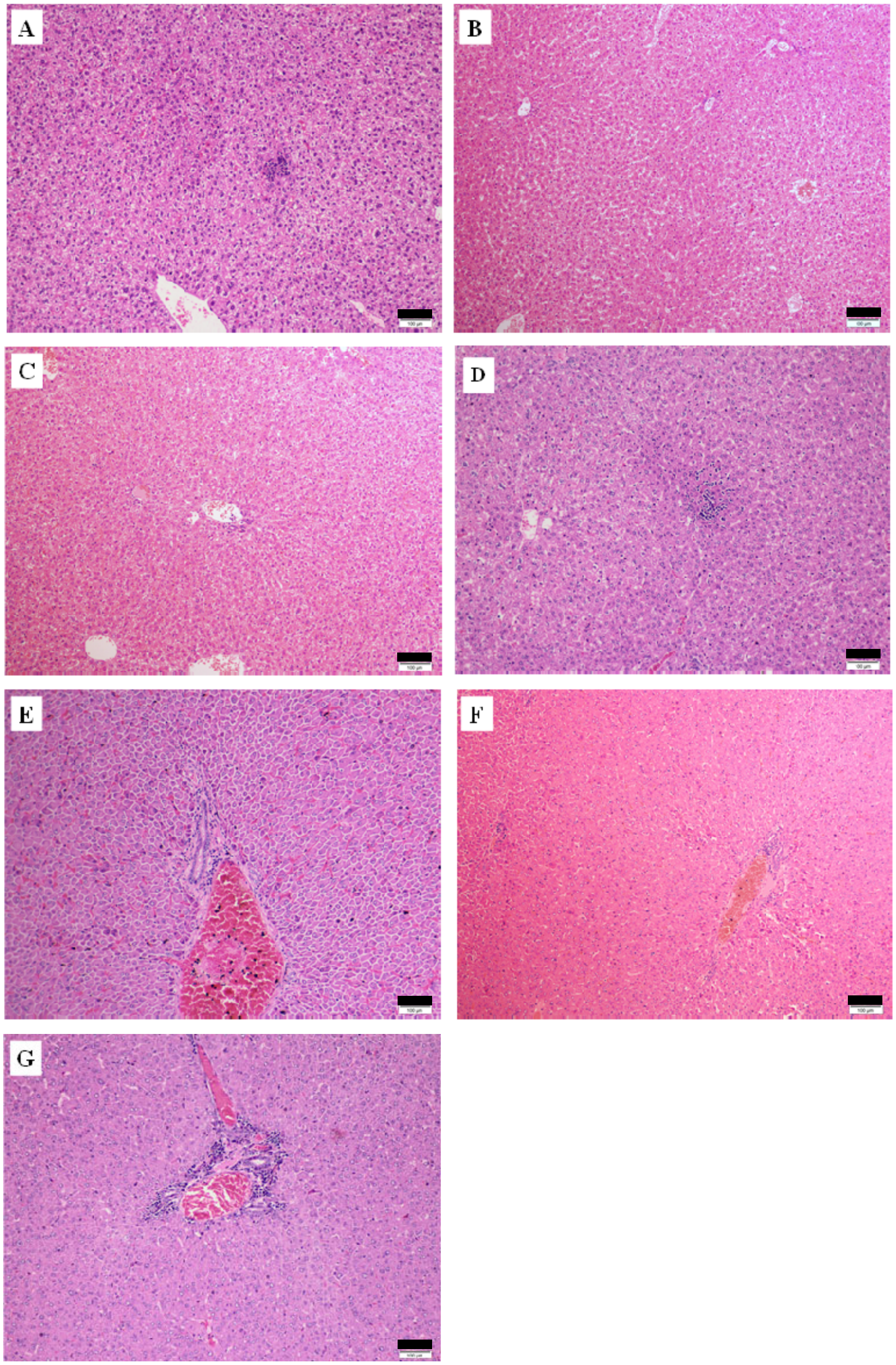

Figure S3. Liver histopathology showing mild inflammation in hamsters vaccinated with various vaccine formulations (Table S3). The micrographs were recorded at 100× magnifications after hematoxylin and eosin staining. (**A**) LMQ (a combination of neutral liposome, monophosphoryl lipid A, and *Quillaja saponaria* fraction 21) adjuvant control; (**B**) Killed whole cell vaccine with Freund's adjuvant; (**C**) Mono-LigAc subunit vaccine with LMQ. (**D**) Mono-LenA subunit vaccine with LMQ; (**E**) Mono-LcpA subunit vaccine with LMQ; (**F**) Mono-Lsa23 subunit vaccine with LMQ. (**G**) Multisubunit

vaccine (containing LigAc, LenA, LcpA, and Lsa23) with LMQ. Black bar: 100 μm.

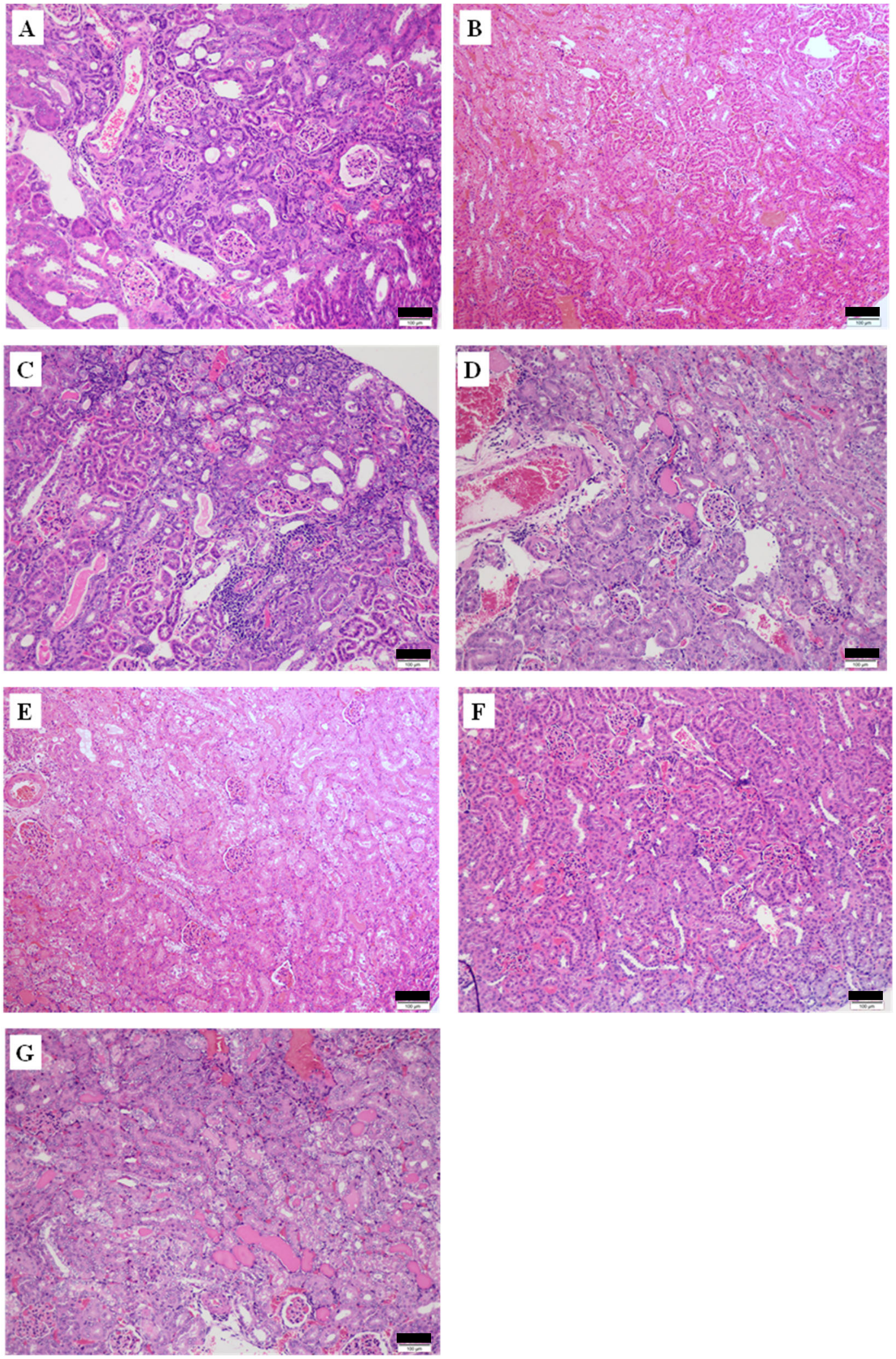

Figure S4. Kidney histopathology showing sparse tubulointerstitial nephritis from hamsters vaccinated with various vaccine formulations (Table S3). The micrographs were recorded at 100× magnifications after hematoxylin and eosin staining; (**A**) LMQ (a combination of neutral liposome, monophosphoryl lipid A, and *Quillaja saponaria* fraction 21) adjuvant control; (**B**) Killed whole cell vaccine with Freund's adjuvant. (**C**) Mono-LigAc subunit vaccine with LMQ; (**D**) Mono-LenA subunit vaccine with LMQ. (**E**) Mono-LcpA subunit vaccine with LMQ; (**F**) Mono-Lsa23 subunit vaccine with LMQ; (**G**) Multisubunit

vaccine (containing LigAc, LenA, LcpA, and Lsa23) with LMQ. Black bar: 100 μm.

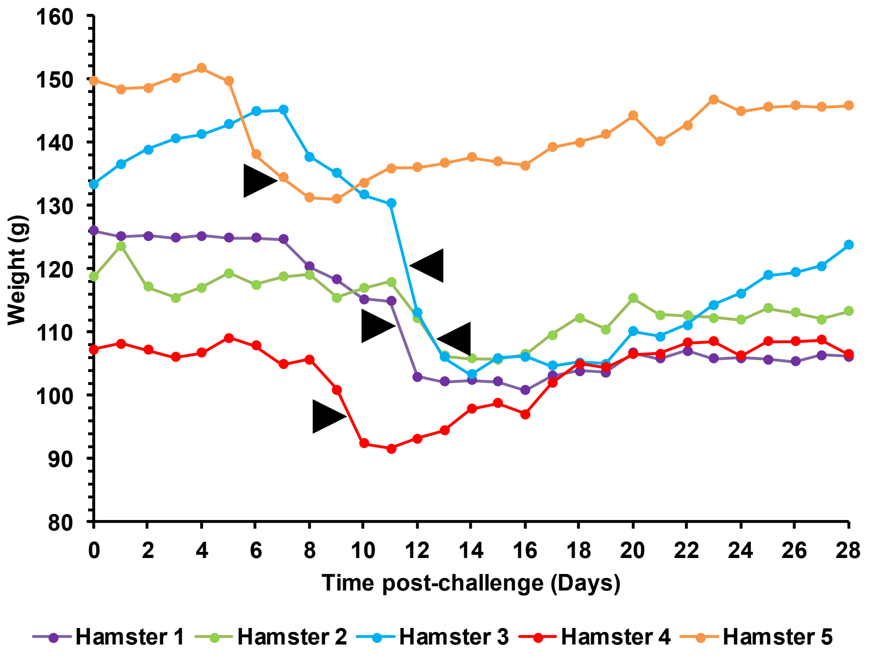

Figure S5. Weight of five surviving hamsters after challenge with virulence *L. interrogans* serovar Pomona. The hamsters were previously immunized with various vaccine formulations. Each vaccinated hamster was challenged intraperitoneally with 20× LD50 of low passage *L. interrogans* serovar Pomona)the same strain used in this study(. The hamsters were weighed and monitored daily for end-point criteria up to 28 days post challenge. Black arrow head indicates 10% weight loss of each hamster.

Summary

Five surviving hamsters with 10% weight loss (black arrow head) could later gain their weight and finally survived up to day 28 post challenge. Therefore, 10% weight loss was not correlated with dead outcome and we used 20% weight loss as one of the end point criteria in this present study.

Table S1. Oligonucleotides used in this study.

Protein	Primer Sequence 5′ to 3′[a]
LigAc	F: ATTCGGATCCTTACCGTTTCCAACACAAAC<br>R: ATTAAAGCTTTTATGGCTCCGTTTTAATAGAG
LenA	F: CCGCATATGCTTTATTCGTGTGGGGAT<br>R: CGGCTCGAGTTACTGTTCTACACAGAGAA
LcpA	F: ATTCGGATCCCTTCGAGGTTGGAAATCGTTTC<br>R: ATTAAAGCTTTCATTTTTCTGGAGGAAGAACG
Lsa23	F: ATTCGGATCCAACTTCCTTACTTTTCCCCTAAC<br>R: ATTAAAGCTTGAATGTTGACTAGAGGCATTTA

[a] Underlining represents restriction enzyme sequences. F: forward, R: reverse.

Table S2. Groups of mice immunized with various vaccine formulations containing recombinant factor H binding protein subunits or controls.

**Antigen**[a]	**Adjuvant**[b]
PBS	LMQ (adjuvant control)
LigAc	LMQ
LenA	LMQ
LcpA	LMQ
Lsa23	LMQ
LigAc, LenA, LcpA, Lsa23	LMQ
PBS	Freund's (adjuvant control)
LigAc	Freund's
LenA	Freund's
LcpA	Freund's
Lsa23	Freund's
LigAc, LenA, LcpA, Lsa23	Freund's

[a] PBS: phosphate-buffered saline; LMQ: a combination of neutral liposome, monophosphoryl lipid A, and *Quillaja saponaria* fraction 21. LigAc, LenA, LcpA, Lsa23: recombinant factor H binding protein subunits.

[b] The final volume ratio of LMQ or Freund's adjuvants to immunogen was 6:4 or 1:1, respectively.

Table S3. Groups of hamsters immunized with various vaccine formulations containing recombinant factor H binding protein subunits or controls.

**Antigen**[a]	**Adjuvant**[b]
Heat-killed whole cell (1x10^8cells)	Freund's
PBS	LMQ (adjuvant control)
LigAc	LMQ
LenA	LMQ
LcpA	LMQ
Lsa23	LMQ
LigAc, LenA, LcpA, Lsa23	LMQ

[a] PBS: phosphate-buffered saline; LMQ: a combination of neutral liposome, monophosphoryl lipid A, and *Quillaja saponaria* fraction 21. LigAc, LenA, LcpA, Lsa23: recombinant factor H binding protein subunits.

[b] The final volume ratio of LMQ or Freund's adjuvants to immunogen was 6:4 or 1:1, respectively.
